# Public Perceptions of Diabetes, Healthy Living, and Conversational Agents in Singapore: Needs Assessment

**DOI:** 10.2196/30435

**Published:** 2021-11-11

**Authors:** Dhakshenya Ardhithy Dhinagaran, Thirunavukkarasu Sathish, Tobias Kowatsch, Konstadina Griva, James Donovan Best, Lorainne Tudor Car

**Affiliations:** 1 Lee Kong Chian School of Medicine Nanyang Technological University Singapore Singapore Singapore; 2 Population Health Research Institute McMaster University Hamilton, ON Canada; 3 Centre for Population Health Sciences Lee Kong Chian School of Medicine Nanyang Technological University Singapore Singapore; 4 Future Health Technologies Programme Campus for Research Excellence and Technological Enterprise Singapore-ETH Centre Singapore Singapore; 5 Centre for Digital Health Interventions Department of Management, Technology, and Economics ETH Zurich Zurich Switzerland; 6 Centre for Digital Health Interventions Institute of Technology Management, University of St Gallen St Gallen Switzerland; 7 Department of Primary Care and Public Health School of Public Health Imperial College London London United Kingdom

**Keywords:** conversational agents, chatbots, diabetes, prediabetes, healthy lifestyle change, mobile phone

## Abstract

**Background:**

The incidence of chronic diseases such as type 2 diabetes is increasing in countries worldwide, including Singapore. Health professional–delivered healthy lifestyle interventions have been shown to prevent type 2 diabetes. However, ongoing personalized guidance from health professionals is not feasible or affordable at the population level. Novel digital interventions delivered using mobile technology, such as conversational agents, are a potential alternative for the delivery of healthy lifestyle change behavioral interventions to the public.

**Objective:**

We explored perceptions and experiences of Singaporeans on healthy living, diabetes, and mobile health (mHealth) interventions (apps and conversational agents). This study was conducted to help inform the design and development of a conversational agent focusing on healthy lifestyle changes.

**Methods:**

This qualitative study was conducted in August and September 2019. A total of 20 participants were recruited from relevant healthy living Facebook pages and groups. Semistructured interviews were conducted in person or over the telephone using an interview guide. Interviews were transcribed and analyzed in parallel by 2 researchers using Burnard’s method, a structured approach for thematic content analysis.

**Results:**

The collected data were organized into 4 main themes: use of conversational agents, ubiquity of smartphone apps, understanding of diabetes, and barriers and facilitators to a healthy living in Singapore. Most participants used health-related mobile apps as well as conversational agents unrelated to health care. They provided diverse suggestions for future conversational agent-delivered interventions. Participants also highlighted several knowledge gaps in relation to diabetes and healthy living. Regarding barriers to healthy living, participants mentioned frequent dining out, high stress levels, lack of work-life balance, and lack of free time to engage in physical activity. In contrast, discipline, preplanning, and sticking to a routine were important for enabling a healthy lifestyle.

**Conclusions:**

Participants in this study commonly used mHealth interventions and provided important insights into their knowledge gaps and needs in relation to changes in healthy lifestyle behaviors. Future digital interventions such as conversational agents focusing on healthy lifestyle and diabetes prevention should aim to address the barriers highlighted in our study and motivate individuals to adopt healthy lifestyle behavior.

## Introduction

### Background

The growing burden of diabetes is a matter of global concern. Among developed countries, Singapore has the second highest prevalence rate, with 1 in 9 people aged between 18 and 69 years having the condition [1]. Singapore’s life expectancy increased from 83.2 in 2010 to 84.8 in 2017; however, when adjusted for time in perfect health (health-adjusted life expectancy), Singapore’s life expectancy dropped to 74.2 [2]. The main contributors to this drop are chronic conditions such as diabetes, hypertension, and high cholesterol levels, which are largely due to unhealthy lifestyle and habits [2]. Approximately 440,000 Singaporean residents (aged 18 years and above) were diagnosed with diabetes in 2014, and this number is expected to reach 1 million by 2050 [3].

Prediabetes is a precursor of type 2 diabetes and is becoming increasingly common. Lifestyle interventions, including promotion of physical activity and a healthy diet, delivered by trained health professionals can reduce the incidence of type 2 diabetes in those with prediabetes [4-6]. However, personalized guidance and attention from expert health care professionals are not feasible or affordable on a large scale. A potentially more accessible alternative to in-person support could be digital initiatives for information and intervention delivery, such as conversational agents. Moreover, health programs delivered over the internet have been successful, as exemplified by web-based interventions to reduce smoking [7] and alcohol intake [8] and to improve sexual health [9], cancer screening [10], physical activity [11], and diet [12]. The ubiquity of the internet makes these programs easily accessible to diverse groups. Conversational agents or chatbots are computer programs designed to mimic human-human conversations using either text or speech. Conversational agent use has advantages such as easy access, the possibility of personalization, greater efficiency, bidirectional interactivity, and a chance to build up a working alliance, highlighting the potential to improve patient care [13]. Applications of conversational agents in health care are gaining traction in a number of medical fields and diverse age groups (from children to older adults). Thus far, they have been employed in health care service provision, chronic disease management, patient education and can be delivered via existing messaging apps, individual apps, or even standalone devices [14,15].

People’s knowledge of diabetes, prediabetes, and the role and impact of healthy living can affect their lifestyle choices and ultimately health [16]. Before introducing a novel method of digital health delivery to a study population, a needs assessment study is required. This process will identify the challenges faced by the population, the essential components they wish to see, and gauge their likely acceptability of a new digital initiative for their health [17]. Evidence on conversational agent use for healthy lifestyle promotion in different settings, including Singapore, is currently limited. Singapore, a technologically savvy country, has a high use of mobile phones and messaging apps, as evidenced by its high smartphone penetration rate of 82% in 2021 [18,19]. Singapore’s Ministry of Health has highlighted the increasing use of conversational agents in health care to tackle issues such as the rising chronic disease burden within an aging population [20]. This situation makes Singapore an ideal candidate for the implementation and testing of novel mHealth interventions, such as conversational agents [21].

### Objectives

To address this need, this study aims to design and evaluate a conversational agent that promotes healthy lifestyle behavior changes for the general population in Singapore. Education and widespread delivery of a healthy lifestyle intervention at the level of the general population can be pivotal in the prevention of diabetes and prediabetes even before individuals get to a stage of high risk. To inform the development of such a conversational agent, we invited members of the public to share their views on and experience of diabetes, prediabetes, healthy living, and digital health interventions, which we report in this study.

## Methods

This qualitative study was conducted between August and September 2019. We invited 20 members of the public to participate in the semistructured interviews. The study was approved by the Nanyang Technological University Ethics Committee (IRB-2018-11-032). All participants read the study information sheet before providing written consent. Specific consent was obtained to record the interviews. This study followed the Consolidated Criteria for Reporting Qualitative Research (COREQ) guidelines [22] (Multimedia Appendix 1).

### Participants and Recruitment

**Figure 1:**
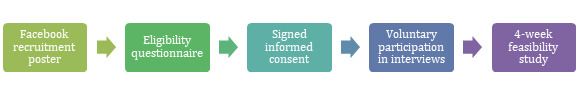
The order of events participants underwent, from (1) recruitment, (2) interviews for needs assessment and subsequent participation in a 4-week feasibility study.

Participants were volunteers initially recruited for a healthy lifestyle change conversational agent pilot feasibility study using a study poster on relevant healthy living Facebook (Facebook Inc) pages and groups (Figure 1). Participants taking part in this study had to read and sign informed consent, complete an eligibility, baseline, and follow-up questionnaire, in addition to conversing with a Facebook Messenger conversational agent over the 4-week study period. We included adults aged ≥21 years who were fluent in English with a Facebook account. The following exclusion criteria were applied:

- individuals with a history of major illness, such as cancer, heart disease, stroke, chronic liver disease, chronic kidney disease, neurodegenerative condition, and hypertension
- individuals with physical disability that would prevent regular physical activity
- pregnant women
- participants younger than 21 years of age
- illiterate or nonwriting individuals (as all questions will be asked in English)

We excluded individuals with a history of major illness, those with physical disabilities, or pregnant women, as advice provided by the conversational agent was not optimized to consider diet and exercise requirements for these populations. In addition, as the content in questionnaires, informed consent, interviews, and conversational agents were all in English, illiterate, and nonwriting individuals were excluded.

We used a purposive sampling strategy to capture variations in ethnicity and age groups from a wider range of perspectives.

### Data Collection

Interviews were conducted by a female PhD student (DAD) in designated private meeting rooms at the Lee Kong Chian School of Medicine, NTU, Singapore. An interview guide was used, and interviews were conducted either in person or over the telephone, depending on the volunteer’s preference. DAD was provided with sufficient details, resources, and exposure to web-based courses on qualitative research and conducting telephone interviews before study commencement. Field notes were taken during the interviews. The initial interview guide was informed by the literature on the development of digital and conversational agent-delivered health interventions (Multimedia Appendix 2) [23-26]. The interview was adapted further as the study proceeded, to take account of emerging themes, and each interview ended when we reached saturation of novel topics (eg, for barriers and facilitators, respectively). The interview was an earlier phase in a larger feasibility study, in which all the participants were enrolled (Figure 1). This study aims to test the feasibility and acceptability of a conversational agent for healthy lifestyle changes in Singapore. Participants were compensated with a digital voucher of SGD $25–35 (US $18-25) upon pilot feasibility study completion, and there was no extra incentive for taking part in the telephone interviews. Participation was voluntary.

Basic demographic data were obtained from all participants, including gender, age, ethnicity, marital status, monthly household income, number and age of children, and occupation.

Their use of conversational agents and feedback on these interactions and suggestions for future health applications (eg, tone, media, and direction) were also explored. These insights would unveil their preferences, which would be useful in directing our design and development of conversational agents for this population. Participants were also asked to share the types of messaging and health apps they had experience using and their opinions on them. Information on their use of messaging apps was important to note, as many conversational agents were delivered via these platforms. Public preferences can direct an appropriate delivery platform for these agents. The naming and patterns of use of health apps would indicate the comfort of the population currently using technology to monitor their health and what applications they find mHealth solutions appropriate for.

Questions were asked about participants’ knowledge of diabetes and prediabetes as well as their family history of these conditions. Identifying their knowledge gaps and awareness of being at risk could help in preventing and reducing the risk of developing diabetes and prediabetes. Finally, to best understand their needs on how to promote healthy living, questions were asked about what they thought were the barriers and facilitators of a healthy lifestyle. The focus was on these topics: eating habits, physical activity, stress, and sleep. Participants were asked about their current habits and thoughts on what the ideal advice for these components should be. This would inform the content of future conversational agents educating the public on healthy lifestyle changes.

Both short-and long-answer questions were asked. An example of a short answer question is *Do you use messaging apps such as Facebook Messenger?* An example of a question that required a more comprehensive answer is *Sometimes we do not always eat the way we would like to. Let’s talk about some difficulties you experience with eating healthy.*

All in-person interviews were conducted using a portable audio recorder. Telephone interviews were conducted via a speaker phone using the departmental iPhone 6 (Apple Inc) and recorded using an audio recorder placed in close proximity to the speaker phone and the interviewer. The interview lasted approximately 30 minutes. The audio files were transcribed verbatim. All transcripts were checked with the corresponding sound files for accuracy, and they were subsequently corrected for errors and cleaned.

### Data Analysis

The data were analyzed using Burnard’s method, a structured approach for thematic content analysis established in 1991 and is still used today [27]. First, 2 researchers familiarized themselves with the transcripts by reading them multiple times. Second, the initial codes were proposed. Third, the themes were derived from the codes. Fourth, the 2 authors discussed and combined their themes for comparison. Finally, they reached a consensus on the themes to be used and how to define them. The coding of transcripts was performed using the *review* and *comments* function on a word processor. The final codebook is presented in Multimedia Appendix 3.

## Results

### Overview

We approached the 60 participants enrolled in the conversational agent feasibility study (via email), looking for 20 volunteers to participate in a telephone interview (Figure 1). We chose this number based on the recommendation that qualitative analysis should be based on data from 1-30 respondents [28]. A total of 20 participants responded and agreed to be interviewed. Seven interviews were conducted in person, and 13 were conducted over the telephone.

### Participant Demographics

Eighty percent of the participants were female, median age was 30 (in complete years), age range was 23-60 years, and 50% (10/20) were married. Of the 20 participants, 65% (13/20) were working, 25% (5/20) were full-time students (undergraduate or postgraduate), and 10% (2/20) were unemployed. More than half of the participants (11/20, 55%) were Chinese, 25% (5/20) were Indian, 15% (3/20) were Malay, and 5% (1/20) was White. The monthly household income was SGD $6000–8000 (US $4500-6000) for 25% (5/20) of the participants, SGD $8000-10,000 (US $6000-7500) for 20% (4/20), SGD $10,000-15,000 (US $7500-11,000) for 20% (4/20), SGD $4000-6000 (US $3000-4500) for 15% (3/20), less than SGD $4000 (US $3000) for 15% (3/20), and more than SGD $15,000 (US $11,000) for 5%(1/20). The median monthly household income in Singapore in 2019 was SGD $9425 (US $7000) [29].

### Thematic Analysis

**Figure 2:**
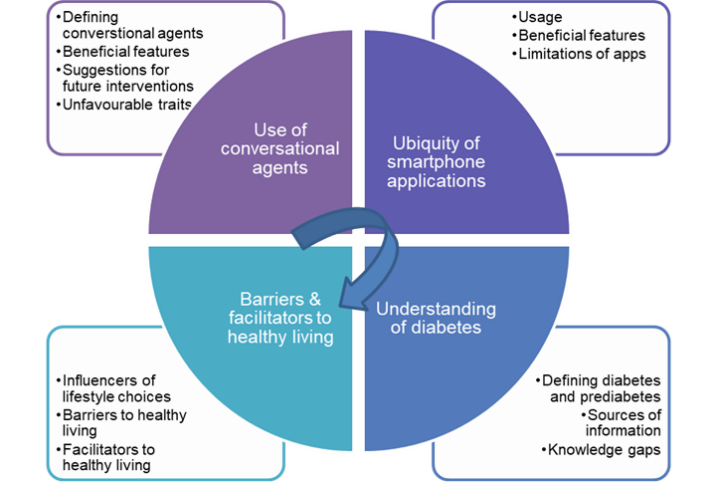
Themes and subthemes identified from analysis of interview transcripts.

Four main themes were identified: (1) use of conversational agents, (2) ubiquity of smartphone apps, (3) understanding of diabetes, and (4) barriers and facilitators to a healthy living in Singapore (Figure 2).

We present each theme below, along with subthemes and excerpts that highlight different facets of the theme.

### Theme 1: Use of Conversational Agents

#### Definition of Conversational Agents

Many participants were able to provide a definition for what a conversational agent was by mainly using the key words *AI programme* or *virtual call centre* with which one can communicate. Participants were also aware that conversational agents were readily available at all times of the day. Existing applications include car insurance, public transport, web-based shopping, banking, and airline services. A couple of participants were unfamiliar with this term.

#### Beneficial Features and Suggestions for Future Interventions

They shared their views on what makes a conversational agent appealing. Conversational agent preferences included accuracy of information, interactivity, visual aids, text, predefined options, a group component, novelty, good reach, incentives, appropriate tone, frequency, duration, and bidirectional communication. Seven participants showed a preference for visual aids (ie, images and videos) as well as text when communicating with the conversational agent.

Predefined options were preferred over AI if the conversational agent was not sufficiently programmed to deal with more complex questions and requirements. Two participants indicated a liking for peer support in a conversational agent intervention to support their behavior change. Some participants wanted novelty to be introduced in the conversational agent’s delivery method to avoid the routine from becoming too monotonous and boring to continue:

But that one was not very good because they cannot... It becomes a pattern. Once they get used to it, then people will try to run away from the pattern.

P15

Novelty was proposed in the timing of message delivery and message format. For example, some may provide educational information or reminders for healthy behaviors and others, a quick tip for the day.

Conversational agents that had a good reach (ie, were relevant to a wide range of age groups) were most appreciated. Participants mentioned that web-based or digital health platforms such as apps and conversational agents would be well received by the younger population. They indicated a need to present all the relevant information, advice, and reminders on healthy living to older populations who may be less tech savvy by employing simple technology or supplementing with nondigital means. Conversational agents that offered incentives in the form of rewards or discounts were also of interest. Two participants explicitly indicated greater interest in using those apps or conversational agents that, for example, provided discounts for grocery shopping, or coupons for redemption.

Participants had varied views on a suitable frequency and duration of interaction, ranging from daily to weekly. The average suggestion from all 20 participants was a 2-3 days a week frequency, for a duration <20 minutes. Participants were generally indifferent to the tone of a conversational agent but did mention that layman terms should be adopted, and generally, a friendly and informal tone was preferred. Bidirectional communication (where the user can respond and talk to the conversational agent) was preferred over unidirectional communication (where only the conversational agent talks and the user consumes information). Nine participants shared that bidirectional communication would give them the option to ask questions to the conversational agent, clarify doubts, or ask for more information on a topic of interest:

I think two way should be an option. In case like there are any questions or anything you might want to follow up with. Or as well as like… you’re interested in something, you can like ask for a contact or they could get you to a website for more information or somewhere that kind of thing.

P05

#### Unfavorable Traits

Participants also shared instances in which conversational agent use was unfulfilling. Five participants had negative experiences in the past, where the conversational agents they encountered were unable to answer their questions or provided irrelevant answers:

When I had questions, they were not answered appropriately. So, I had to resort to calling them.

P09

Others indicated that sometimes they were unsure of the conversational agent’s level of intelligence and realized through the interaction that the agent was not sufficiently artificially intelligent to address their more complex questions. In such cases, they would then prefer a simpler agent (with predefined options) that could fulfill its tasks more seamlessly.

### Theme 2: Ubiquity of Smartphone Applications (Specifically Messaging and Health Apps)

#### Use of Mobile Apps

As conversational agents are frequently embedded in existing messaging apps or implemented as standalone apps, we explored participants’ use and opinions of messaging and health apps. All participants mentioned that they used messaging apps, and 17/20 (85%) used health apps. The messaging apps used were WhatsApp (Facebook Inc), Facebook Messenger (Facebook Inc), Telegram (Durov Software), WeChat (Tencent Holdings Limited), and LINE (Naver Corp). These were used mainly for communicating and socializing, sometimes in large groups. Some use it as a means of sharing information for educational and professional purposes.

A variety of health apps were mentioned with *Healthy 365* and step trackers were the most frequently used. Other apps were MyFitnessPal, AIA vitality, Fitbit, Samsung Health, Health Promotion Board app, Nike Run Club, Chronometer (monitor nutrition), and Stride (habit tracker).

#### Beneficial Features

Specific smartphone app features and functions that make apps more favorable for use were discussed. Eight participants attributed the beneficial features of these apps to fulfilling a necessity, ease of use, engagement (promoted by the availability of stickers), and their multifunctionality, such as texting, calling, and video calling.

Something else participants shared which promotes smartphone app use, is the provision of incentives:

And then for the Healthy 365 app by HPB (Health Promotion Board is a Singapore Government organisation), I use it to redeem the rewards and participate in their promotional activities and programs.

P17

#### Limitations of Apps

Two participants reported that sometimes the content and frequency of messages could be too overwhelming to deal with*.* The accuracy of the app content was sometimes questioned by participants (2/20, 10%), with different apps reporting different numbers for the same measure.

### Theme 3: Understanding of Diabetes

We explored the participants’ understanding and knowledge of diabetes, prediabetes, and the sources of information that they used to obtain relevant information. Understanding their current level of knowledge and what information they need more exposure to could be incorporated into a healthy lifestyle conversational agent to prevent diabetes and prediabetes in the general population.

#### Diabetes

All participants were able to provide a definition of diabetes, some more comprehensive than others. Five individuals mentioned insulin or the inability of the body to control their blood sugar level, which is too high. Four participants stated that diabetes requires one to reduce sugar intake.

They were also aware that there may be a need to take medication and that there is no cure for diabetes. Participants shared the impact of diabetes on their health. Five participants were aware that diabetes can affect other parts of the body and lead to other illnesses, including kidney failure, stroke, high blood pressure, and limb amputations.

Regarding impact on one’s life, participants mentioned that diabetes affects families, ability to exercise, and breathing, and can lead to higher medical expenses:

Having good health to be drained away. And then, how does this affect families concerned...I was told that people with diabetes may not be able to exercise actively. Have to change the type of exercise that they do. Sometimes breathing will be affected, also.

P14

#### Prediabetes

Fifteen participants were able to define prediabetes, stating that it was a condition where someone is at a borderline risk of developing type 2 diabetes. Two individuals reported that prediabetes was reversible:

Basically, you are at risk of diabetes if you don’t change the way you live your life, or your diet.

P20

Other participants were very unfamiliar with the term *prediabetes* (5/20, 25%) and acknowledged that diabetes was more commonly talked about:

I always saw this like pretype or something number 2 or something like that so I don’t really understand it as well

P08

#### Sources of Information

The sources of information that they used to learn more about diabetes varied. Some shared that their personal experiences with diabetic family members helped them to be educated on diabetes and its implications (2/20, 10%):

And you know when she (mother) was around, there’s a diabetes society or something we used to go and visit, attend talks and yea, somewhere in(…) it’s quite near my place actually but after she passed away uh I’m not involved in anything anymore

P01

Others mentioned the government’s role in educating the public on diabetes, prediabetes, and healthy living and how the government can further influence the public in the near future (3/20, 15%):

But from what I used to experience, the government does have certain policies acting through MOE (Ministry of Education). For example, last time we used to have no fried food in canteen. I suppose they must be doing something with school canteens because of the diabetes.

P17

In addition, participants mentioned that a dearth of sufficient information was readily available to them, causing a barrier to awareness on healthy living (6/20, 30%). Some participants felt that the older generation may have less information because of lower levels of technological competency in searching for information on the web.

Participants proposed additional means to improve information availability and population awareness. Some of these methods include advertisements on the television and internet, as well as a display of the number of calories for each food item at hawker centers (food courts with affordable local meals):

and also there could be more awareness like you know when we go to restaurant or maybe hawkers more information could share like the calories. Because yea I know some hawker centres they do show but it didn’t exactly show the number of calories for all the food.

P08

#### Knowledge Gaps

Participants identified some areas where they thought societal understanding was low and should be addressed in the future. One such example was more information on physical and psychological changes for a healthier lifestyle:

What the individual lifestyle changes that they can make or consider to make.

P12

Yes. Definitely. I think when you are more stressful, then your sugar goes up too.

P20

Mental health.

P15

They made specific references to education on the importance of healthy eating and physical activity. One participant felt that instead of learning more, it was more important to apply what is already known about diabetes.

Further information on symptoms, prevention, risk factors, and awareness of being at risk were of interest. In addition, there is a need for more information on the implications of diabetes, its management, and sources of support:

Maybe even things like prognosis if you are diagnosed, how are you going to manage it. If they need help, who can they look for.

P11

### Theme 4: Barriers and Facilitators to a Healthy Living in Singapore

#### Influencers of One’s Lifestyle Choices

Participants shared their existing lifestyle habits and the factors that enable or hinder healthy living. These barriers are helpful indicators of what a future conversational agent can aim to address when administering advice, education, and support for healthy lifestyle changes.

They first discussed the relationship between their existing lifestyle choices and how this relates to their perceptions of current body weight. Some participants reported dissatisfaction with their current weight. They perceived themselves as either too heavy (9/20, 45%) or underweight (2/20, 10%).

Participants also highlighted the existing factors which drive their lifestyle choices.

##### Convenience

Convenience was a big factor. This was relevant regarding food available in the close vicinity, as well as the convenience of having a gym or group exercise class easily accessible to them.

##### Cost

Cheaper food was preferred because of its affordability, over more expensive, healthier options. Participants also indicated a desire to engage in group exercise sessions, but only if they were affordable:

The biggest problem we have here is the price of healthy food. It’s just infinitely more costly. For example, some salad can cost you six, SGD $7 (US $5), whereas chicken rice can cost you SGD $3 (US $2). You cannot possibly eat salad every day even if you are earning okay. But if you are one of those that are low income, definitely you will go for three-dollar chicken rice every day. And in the long run, it just causes a lot of problems.

P17

Maybe I'd join Zumba. I like that kind of thing but I'm thinking about affordability and the convenience.

P18

##### Personal Preferences

Some participants chose to eat food based on their personal preferences (5/20, 25%). Similarly, they chose to engage in physical activities that they enjoyed or suited their schedules:

Because I know that I’m not really hectic with the exercising, so I would tend to think that my house is three bus stops away from the interchange, so I will tell myself let’s just walk from the MRT back home, so at least it’s some walking activity for me.

P18

##### Social Activities

Some participants chose what to eat based on the kinds of social activities they engaged in and the food available there (3/20, 15%). Furthermore, their social life was influenced by the amount of exercise they could do as more time spent socializing left less time for physical activity (2/20, 10%).

##### Advertising and Marketing

Marketing of food was a factor influencing the kind of food they decided to eat in a day (2/20, 10%):

It just depends on what I feel like eating or maybe a bit of marketing as well. For example, if McDonald’s has a new menu, then I might just go for it

P17

##### Nutritional Value

Nutritional value of food determined whether participants chose to eat it:

If I look at the menu item and then it looks like this is 600 calories, which is top of the list out of the whole list of things that I’m looking at, I would think twice about eating it

It does have some sort of impact knowing that you are intentionally consuming so much. You will trigger some automatic response to self-regulate.

P17

##### State of Mind

Some participants mentioned that their mood or state of mind largely determined the food they decided to consume (4/20, 20%). For example, when upset, stressed, or in a bad mood, they gravitated toward food that was more comfortable than healthy. Likewise, their state of mind also influenced their physical activity (3/20, 15%):

like after a long day of work, I’m not like oh I’m going to go to the gym you know? Like nah I just want to stay at home and relax.

P05

#### Barriers to Healthy Living

Participants shared some barriers to healthy living.

##### Dining Out

Having to dine out on a regular basis meant that participants had no control over the nutritional value of their food:

Sometimes if you like to go to a restaurant and you hope that there’s not so much MSG (monosodium glutamate–flavour enhancer) its really beyond your control to tell the chef hey I don’t want more… I don’t want the MSG or I want less salt. I mean you can tell them less salt. But pre-prepared stuff usually its hard.

P06

##### Lack of Appeal

Two participants mentioned that healthy foods tend to have an unappealing taste and texture, which discouraged its consumption:

Eating healthy, sometimes, they say is very bland, very tasteless. No fried is something without aroma. So, eating healthy sometimes lack of the aroma. And then they say it’s either very too liquid, or so tasteless, that is the thought they have.

P14

##### Health Condition

One participant shared that a health condition (brain tumor) brought about compulsive eating habits, which caused her to deviate from a healthier diet.

##### Fear of Injury

Two participants also mentioned a fear of injury or internal damage to their bodies, which may be caused by physical activity, preventing them from exercising. Hence, this fear became a barrier to a more active lifestyle:

but there is also a fear that at a certain age there is some wear and tear in the body.

P14

##### Lack of Free Time

Long working hours were associated with less free time and, hence, less frequent exercise. This lack of work-life balance also exacerbates stress:

whether I have time to dock off for a run or not. It really depends because most of the time, I’m actually working quite late…. I think for myself would definitely be work stress…. So myself sometimes I work 15 to 16 hours a day.

P08

##### Specific Events

Specific events, such as unexpected incidents, can disrupt an individual’s equilibrium and cause stress. Similarly, events such as exams can negatively impact sleep schedules. Changes in weather, such as a hazy or wet period in Singapore, can prevent individuals from being physically active if there is a preference to exercise outdoors.

##### Lack of Knowledge

Lack of knowledge was mentioned by several participants (11/20, 55%) as a barrier to healthy living. For example, some participants had trouble going to bed early and did not know how to fix this problem. Others were unsure about how to manage their stress, and hence, the problem persists until they find or learn a solution:

I’m having a bit of difficulty here because I’m experiencing a new sort of stress after coming back from my maternity leave. So, that’s why I don’t really know what to tell you because I think I haven’t really learned how to manage the stress.

P13

#### Facilitators of Healthy Living

Participants shared some factors which can help to promote healthy living.

##### Early Intervention and Knowledge Building

Building knowledge on healthy living from a young age was stated as a promoter. Building more as an adult on existing knowledge of the importance of healthy behaviors could positively influence individuals.

##### Reminders

Although the knowledge may already be there, reminders were cited as a way to reiterate healthy eating behaviors. Participants also requested education on calorie counting, which would promote healthier eating:

So, maybe when it comes to before lunchtime, two hours, tell them this is a suggested meal, then it’s a guideline. Because everybody like to hold their phone, and then the message pops up, and they say, oh, yes, why not, I go and try.

P14

##### Incorporate Activity Into Existing Routines

Sourcing out alternative methods of reaching the recommended weekly level of physical activity and how to be more active in general were found to be helpful. These came in the form of efficient exercising or reducing the amount of time spent sitting after a meal:

So, yes, I only exercise 15 minutes each time. But I try to keep it more vigorous. So, yes, I do HIIT kind of training. So I do that about four or five times a week maybe. I think that there’s more yield in doing vigorous exercise over moderate exercise. So in terms of the efficiency, the amount of time you need to spend, so I try to do more of HIIT.

P20

##### Empowering Oneself

Empowering individuals to provide services for themselves as much as possible—for example, making homemade food was suggested to be an effective way of eating healthier. Engaging in exercise at home and setting up their own workouts were also mentioned. Similarly, healthy self-management of stress in the form of therapeutic activities, for example, was cited as a good way to maintain a healthier lifestyle:

So I have a treadmill at home and I use that. Like I walk as I’m doing some work on my laptop. So I try to incorporate that. Or I might do maybe like 10/15 minutes of yoga or cardio or something just around the house yea wherever I can.

P05

##### Group Support

It was proposed by 4 participants that group support, which evokes a collaborative spirit could encourage more physical activity:

If I did it with other people, like with friends and stuff yea, you could make it like a group effort. Then it’ll be easier because then there are other people to monitor you, just for yourself.

P05

##### Internal Motivators

Internal motivators, such as wanting to be healthy in the present and future, encouraged individuals to take ownership and eat healthily.

##### Discipline

Enforcing some discipline with regard to eating on time, drinking enough water, and planning meals for the week were all suggestions for promoting a healthier diet:

But personally, I do my own meal prep actually. So, I cook for three days, so three of the lunches is covered for me.

P11

Having good discipline and planning slots for physical activity were mentioned as effective methods for developing a more active lifestyle. Discipline in developing and sticking to a bedtime routine that involves winding down, relaxing, and reducing screen time as well as cutting down caffeine intake were proposed by participants to improve sleep habits. Recommendations for disciplining food intake through moderation involved a balance of healthy and unhealthy foods, controlling portion sizes (overall quantity and food pyramid recommended portions), and restricting unhealthy food items. These choices were cited as effective promoters for healthy living:

For example, bubble tea right, everybody likes bubble tea, so to me I will limit myself to buy that maybe once every two months, take that as a reward for myself, yes, that’s what I’d do.

P18

##### Moderation

One participant recommended establishing moderation at work to have a more work-life balance to reduce stress levels:

And I think having a good balance, life, social life, helps as well, that it’s not just work.

P11

Participants also mentioned compensating for sleep over the weekends to moderate overall sleep quantity.

## Discussion

### Principal Findings

To our knowledge, this is the first qualitative study to investigate the public’s perception of conversational agents for a healthy lifestyle change intervention in Singapore.

Participants’ perceptions of conversational agents were discussed, where they offered definitions, usage experiences, and preferential features. These are pivotal in informing the design elements for a feasible and acceptable conversational agent initiative in a health care setting in Singapore. Participants shared their views on the ubiquity of smartphone apps, whereby messaging apps were a necessity for daily communication, social, educational, and professional purposes. This is an important point, as conversational agents can be embedded in messaging app platforms that are most familiar to the target population for easy adoption. Health apps were used for a variety of purposes such as step tracking, health tracking (nutrition, water intake, and habit tracking), or pairing with a Fitbit watch. The fairly diverse patterns of use indicate an inclination to adopt mHealth solutions for numerous purposes. Furthermore, participants’ views on the beneficial and limiting features of apps and conversational agents are highly relevant and should be considered when developing a health conversational agent for this population. Participants shared their existing knowledge on diabetes, prediabetes, and their existing sources of information. In addition, they offered their views on what constitutes healthy living with a focus on diet, exercise, sleep, and stress as well as thoughts on the barriers and facilitators of healthy living in Singapore. The culture of eating out in Singapore was said to be fueled by affordable hawker centers, fast food, and fast-paced life, which does not allow much time for food preparation at home. Frequent dining out, high stress levels, lack of work-life balance, and lack of free time to engage in physical activity were some of the common complaints hindering a healthier lifestyle. In addition, work-related stress has been implicated in poor sleep quality and quantity. Participants proposed that these barriers could be tackled with some self-discipline, preplanning, and sticking to a routine for healthier living patterns. Given the ubiquity of smartphones, the avid use of messaging apps and an inclination to use mHealth initiatives and awareness of these barriers and facilitators for healthy living could be communicated to the population via a conversational agent.

### Comparisons With Existing Literature

Lim et al [30] explored the barriers and facilitators of healthy eating in Singapore in 2019. This study noted that components that encouraged healthy eating included self-discipline, fear of disease complications, education by a health professional and mass media, and health promotion campaigns [30]. Participants in our study also mentioned self-discipline as a strong facilitator and acknowledged the presence of health promotion campaigns but did not cite these as influencers of their lifestyle choices. Furthermore, fear of disease complications and education by health professionals was possibly more relevant in Lim et al [30], as participants were recruited from polyclinics and were already diagnosed with prediabetes. This also points out the need for a more hybrid approach when it comes to more high-risk populations (such as those with prediabetes), as HCP involvement would be necessary in addition to a conversational agent. Furthermore, the lack of skills to prepare and choose healthy foods was shared as a difficulty in healthy eating [30]. This barrier was also noted as a limitation to a healthy diet in our study. Participants added that a lack of time to do grocery shopping, prepare and cook meals was due to their hectic schedules, making dining out a more convenient, but often less healthy, compromise.

Another study noted that healthy eating and physical activity were the main preferred components for health education and communication in patients with prediabetes in Singapore [31]. A similar outcome was implicated in our study, but we also considered the components of sleep and stress for a more holistic view of healthy living. Other necessities for health communication were risk and prevention of diabetes [31]. These were also included in the qualitative analysis. In addition, participants shared an interest in information on where to go for help if they were at risk or diagnosed with prediabetes or diabetes.

Other existing studies have investigated novel conversational agent interventions, with some reporting on the acceptability and usability of these agents. However, an extensive needs assessment analysis, such as the one presented here, for a conversational agent intervention has not been performed so far in the Singaporean population. A scoping review of conversational agents showed that qualitative data were presented in some studies to show the acceptability and satisfaction of these interventions, namely, participants’ opinions of an already developed intervention [14]. These parameters were often reported with Likert scale ratings, but not comprehensively or thematically [32,33]***.***

In a study on behavior change in overweight adolescents, high compliance to conversational agent intervention was attributed to a rewarding game system [34]. This finding aligns with the suggestions from participants in this study that incentives will be well received and will contribute to their willingness to use the conversational agent. Other studies have examined the use of conversational agents for chronic conditions, including diabetes, and reported a preference for the features of conversational agents that allowed for personalization [35]. Personalization was also noted as an advantageous trait in our study participants.

Ta et al [36] discussed the type of support provided by a popular chatbot available on app stores, *Replika*, with generic capabilities such as stress management and social support. Based on the analysis of public user reviews and a survey conducted with participants recruited via social media in the United States, companionship was the greatest form of social support users identified. They attributed this to Replika’s ability to use a range of message types (eg, text and images) and appear human-like. Similarly, interviewees in our needs assessment supported the use of visual aids in addition to text. Although they did not indicate specific personality preferences or the need for the conversational agent to be human-like, a general inclination toward a friendly and informal tone was identified.

Studies on physicians’ perceptions of conversational agents have indicated their apprehension toward agents that are unable to comprehend the emotional state of vulnerable patients and have dissuaded their use in cases where expert medical knowledge and skills are required [37]. However, they have supported the use of conversational agents to support, motivate, and coach patients. In the same way, we aim to develop a conversational agent to support a healthy lifestyle change by understanding the needs and preferences of prospective users via this needs assessment.

### Implications for Future Work

Future digital interventions aimed at encouraging diabetes prevention in the general population to promote and prolong healthy living can incorporate the content-and delivery-specific options highlighted in Textbox 1.

These suggestions include improving knowledge in areas directly relevant to diabetes prevention, building up an awareness of risk factors, symptoms, and prevention methods, as well as the complications and consequences of diabetes and prediabetes. Future applications can also explore expanding actionable advice on how to apply the knowledge participants acquired to healthy living.

It would be beneficial to help users improve their skills related to food choices, calorie counting, and stress management. Advice on healthy eating should be mindful of the Asian diet that most Singaporeans are familiar with and have a preference for. In relation to calorie counting, the usefulness of sharing information on the number of calories in frequently consumed food items in Singapore hawker centers should be examined. Stress management techniques relevant to establishing a work-life balance would potentially be beneficial, as this is a common complaint in Singapore, as disclosed by our participants. In addition, teaching participants time management skills may help to reduce overall stress, providing time and motivation for physical activity, meal preparation, and a bedtime routine. Again, the outcomes of this intervention would need to be evaluated.

Options to reach the weekly recommendation for physical activity, such as increased efficiency, safe exercise options, or suggestions to see a doctor for more personalized advice can be made via a digital conversational agent. Regarding group exercise, cheaper options or even web-based options may be listed. This approach comes with the added benefits of convenience (no traveling) and affordability. Recommendations on alternative methods of exercising, which do not require participants to be outdoors, could be suggested to overcome barriers related to the weather, such as home workouts. A conversational agent could also reinforce the existing promoters of physical activity, for example, reminding them of the feelings of contentment they will experience.

Participants in our study also provided informative recommendations for the delivery of future digital health interventions, focusing on healthy lifestyle behavior changes. App or conversational recommendations are proposed. These factors have enhanced user experience in the past. They also shared the components they wish to see and would enjoy using in future applications.

Future developments should investigate personalizing the conversational agent to the user’s preferences (timings, advice, etc), enabling easy use, making the exchange engaging, and introducing some novelty to boost user experience. The tone, personality, and language used by the conversational agent should also be adapted to suit the target population (eg, being friendly and using language with a level of complexity familiar to the target population). Some additional considerations, which could be included, are incentives, such as rewards or discounts for groceries, and group support, as participants indicated a preference for these.

On the basis of this study, we are developing a rule-based conversational agent pilot intervention that incorporates recommendations from the participants, including educational information on the definitions and implications of diabetes and prediabetes, as well as actionable advice on healthy eating, physical activity, stress management, and healthy sleep patterns. All the information to be included in the intervention will be evidence-based. We will also incorporate visual aids to supplement the text, and preprogramed options will be provided for the user to choose from, to introduce some degree of bidirectional communication.

**Recommendations on content and delivery for future conversational agent interventions for healthy lifestyle behavior change.**

**Content and delivery for future conversational agent interventions**

- Content
  Furthering knowledge on:
    Implications of diabetes and prediabetes
    Awareness of being at risk
    Symptoms and prevention methods
  Actionable advice on:
    How to apply the knowledge gained on healthy living
  Further development of skills relating to:
    Eating healthily
    Calorie counting
    Options available for meeting the weekly recommendation for physical activity
    Stress management
    Time management
  Type of content:
    Novel—up to date
    Trustworthy—evidence based
- Delivery
  Personalization of content
    Advice on how to incorporate exercise into one’s specific schedule, availability of facilities and preferences
    Habit formation – how to discipline your healthy eating, drinking water, physical activity and bedtime routine
  Suitable frequency and duration
    Ask participants for their preference
    Offer options for participants to choose from
  Easy use
    Predefined options for better efficiency if AI is not yet maximized
  Engaging
    Use stickers and visual aids
    Make the conversation bidirectional so the user can be part of the exchange and can ask questions if necessary
  Relevant to target population
    Tone, personality and language used by the conversational agent to be easily understood, relevant and appropriately tailored for the target population
  *Additional considerations to keep users keen and engaged in the interventions
    Incentives, such as rewards and vouchers
    Group support, for example, chat channel for participants to communicate with others using the same conversational agent to share advice and motivate each other

### Strengths and Limitations

In our study, we recruited and interviewed a sample of 20 participants who were ethnically diverse and covered a wide age range (23-60 years). The collected data were coded in parallel by 2 researchers, with the development and application of a common coding framework through a series of discussions. Our findings may also be applicable to other high-income or technologically savvy Asian countries. Although 20 participants were adequate for this analysis, we would aim for a larger sample size in future studies for greater validity and generalizability.

We extrapolated the focus of this study from healthy living to diabetes and prediabetes prevention. To this regard, we managed to provide suggestions on healthy living not only in the context of wellness but also as a means of preventing conditions of great burden in Singapore—diabetes and prediabetes.

There is a possibility of recruitment bias. The participants were all from Facebook groups and pages with an interest in healthy living. Their opinions may be somewhat skewed as they already came with some knowledge and interest in a healthy lifestyle and some technological competency. In addition, despite our purposive sampling, 16/20 (80%) of our participants were female because of the interest shown by the study volunteers. Both of these reduce the generalizability of the findings as they may not be entirely representative of the Singaporean population at large.

### Conclusions

The participants provided valuable insights on their existing knowledge and sources of information on healthy living and diabetes. They also shared their current patterns of use of messaging apps, health apps, and their views on potential conversational agents, which have not yet been used for health care purposes in Singapore.

In addition, they offered opinions about the importance of healthy living and diabetes prevention, likely to be reflective of a significant part of the at-risk population. Preferences for conversational agents and use of smartphone app were also discussed. Finally, they shared views on barriers and facilitators of healthy living. Our findings can be used to inform the development of future conversational agent interventions and similar mHealth initiatives that target healthy lifestyle behavior changes.

## Appendix

Multimedia Appendix 1 Consolidated Criteria for Reporting Qualitative Research (COREQ): 32-item checklist.

Multimedia Appendix 2 Interview guide.

Multimedia Appendix 3 Codebook from qualitative analysis.

## Abbreviations

AI: artificial intelligence
CORQ: Consolidated Criteria for Reporting Qualitative Research
mHealth: mobile health
