# Supplementary material for: Public Perceptions of Diabetes, Healthy Living, and Conversational Agents in Singapore: Needs Assessment
Source: JMIR Form Res. 2021 Nov 11;5(11):e30435. doi: 10.2196/30435 (PMC8663498; doi:10.2196/30435)
Supplement: Multimedia Appendix 1 [file formative_v5i11e30435_app1.docx]

Multimedia appendix 1: COREQ 32-item checklist

| **Item** | **Guide questions/description** | **Reported on page (#)** |
| --- | --- | --- |
| Domain 1: Research team and reflexivity |  |  |
| 1. Interviewer/facilitator | Which author/s conducted the interview? | 3 |
| 1. Credentials | What were the researchers’ credentials? | 3 |
| 1. Occupation | What was their occupation at the time of the study? | 3 |
| 1. Gender | Was the researcher male or female? | 3 |
| 1. Experience and training | What experience or training did the researcher have? | 3 |
| 1. Relationship with participants established | Was a relationship established prior to study commencement? | No |
| 1. Participant knowledge of the interviewer | What did the participants know about the researcher? | 3 |
| 1. Interviewer characteristics | What characteristics were reported about the interviewer/facilitator? | 3 |
| Domain 2: Study Design |  |  |
| 1. Methodological orientation and theory | What methodology orientation was started to underpin the study? | 2, 3 |
| 1. Sampling | How were participants selected? | 2, 3 |
| 1. Method of approach | How were participants approached? | 2 |
| 1. Sample size | How many participants were in the study? | 2 |
| 1. Non-participation | How many people refused to participate or dropped out? Reasons? | None |
| 1. Setting of data collection | Where was the data collected? | 3 |
| 1. Presence of non-participants | Was anyone else present besides the participants and researchers? | 3 |
| 1. Description of sample | What are the important characteristics of the sample? | 4 |
| 1. Interview guide | Were questions, prompts, guides provided by the authors? | 3 |
| 1. Repeat interviews | Were repeat interviews carried out? | No. |
| 1. Audio/visual recording | Did the research use audio or visual recording to collect the data? | 3 |
| 1. Field notes | Were field notes made during and/or after the interview? | 3 |
| 1. Duration | What was the duration of the interviews? | 3 |
| 1. Data saturation | Was data saturation discussed? | 3 |
| 1. Transcripts returned | Were transcripts returned to participants for comment and/or correction? | No. |
| Domain 3: analysis and findings |  |  |
| 1. Number of data coders | How many data coders coded the data? | 3, 4 |
| 1. Description of the coding tree | Did authors provide a description of the coding tree? | 4, multimedia appendix 3 |
| 1. Derivation of themes | Were themes identified in advance or derived from the data? | 3, 4 |
| 1. Software | What software, if applicable, was used to manage the data? | NA |
| 1. Participant checking | Did participants provide feedback on the findings? | NA |
| 1. Quotations presented | Were participant quotations presented to illustrate the themes/findings? Was each quotation identified? | 5-9 |
| 1. Data and findings consistent | Was there consistency between the data presented and the findings? | 5-9 |
| 1. Clarity of major themes | Were major themes clearly presented in the findings? | 5-9 |
| 1. Clarity of minor themes | Is there a description of diverse cases or discussion of minor themes? | 5-9 |
