# Supplementary material for: Public Perceptions of Diabetes, Healthy Living, and Conversational Agents in Singapore: Needs Assessment
Source: JMIR Form Res. 2021 Nov 11;5(11):e30435. doi: 10.2196/30435 (PMC8663498; doi:10.2196/30435)
Supplement: Multimedia Appendix 2 [file formative_v5i11e30435_app2.docx]

## Appendix D. Telephone interview guide (baseline co-design)

**Venue:** Telephone interview

**Language:** English

**Aim:** To elicit participants views on pre/diabetes-related information needs, preferences and the use of mobile phone messaging to encourage behavioural change

**Format:** a semi-structured interview & survey after participants are recruited and have signed an informed consent

1. **Questionnaire (interviewer-administered)**

Can I first ask you a few questions about yourself? Thank you.

| **Gender:** | ☐ Male | ☐ Female |
| --- | --- | --- |

| **Age:** | 25 – 30 years old |  | **Ethnicity:** | Chinese |  |
| --- | --- | --- | --- | --- | --- |
|  | 31 – 35 years old |  |  | Malay |  |
|  | 36 – 40 years old |  |  | Indian |  |
|  | 41 – 45 years old |  |  | Others (Please specify): |  |
|  | 46 – 50 years old |  |  |  |  |
|  | 51 – 55 years old |  | **Marital Status:** | Single |  |
|  | 56 – 60 years old |  |  | Married |  |
|  | >60 years old |  |  | Others (Please specify): |  |

| **Monthly**  **Household Income:** | below $4,000 |  | **Family Structure** |  |
| --- | --- | --- | --- | --- |
|  | $4,000 – $6,000 |  | No. of children & age(s): |  |
|  | $6,001 – $8,000 |  | No. of dependents: |  |
|  | $8,001 – $10,000 |  | Type of dependency: |  |
|  | $10,001 – $12,000 |  | Partner’s Job profession: |  |
|  | $12,001 – $14,000 |  |  |  |
|  | >$14,000 |  |  |  |

**What do you do for living?**

**Do you have prediabetes?**

**If yes, how did you learn you had prediabetes?**

**Does anyone else in your family have prediabetes or diabetes? If yes, who?**

**Do you have any questions on prediabetes or diabetes?**

**Do you use messaging apps such as Facebook Messenger? Which ones?** **What is your opinion on them?**

**Do you use any mobile applications on prediabetes or diabetes? If yes, which ones? What is your opinion on them?**

**Are you familiar with chatbots? Please explain what a chatbot is.**

1. **Questions on care gaps as well as barriers and facilitators to a healthy lifestyle**

I will now proceed to ask you a few questions in relation to healthy lifestyle and your views on it.

**(Pre)Diabetes knowledge/awareness**

Diabetes is a serious condition that affects many people in Singapore

1. Suppose you had one minute to explain to someone what diabetes is, what would you say?
2. What do you think is the most important topic for you to learn more about to prevent diabetes?
3. Pre-diabetes affects more than 20% of Singaporeans. How would you describe pre-diabetes?
4. If you had pre-diabetes how do you think it would affect your life? If you had diabetes how do you think it would affect your life?

**Eating habits**

1. How would you describe healthy eating habits?
2. Sometimes we do not always eat the way we would like to. Let’s talk about some difficulties you experience with eating healthy. What influences the foods that you decide to eat?
3. What could help you have/maintain healthier eating habits?
4. If someone asked you to suggest how to have a healthier diet, what would you suggest?

**Physical Activity**

1. When you hear the word physical activity what comes to mind?
2. What influences how physically active you are each day?
3. If you do feel you need to get more physical activity, how would you add more physical activity into your daily life?

**Weight management**

1. How do you feel about your body weight?
2. What are some things that you feel would help you achieve or maintain a healthy body weight?

**Stress/sleep**

1. What role does stress have in your life?
2. What would help reduce stress levels in your life?
3. How are you sleeping habits?
4. What would help you improve your sleeping habits?

**mHealth intervention**

I would now like to talk to you about our intervention. We are putting together a diabetes prevention program for people at risk of diabetes. This intervention will be delivered via mobile phones using messages.

1. What are some topics you would want us to include in this interventions?
2. What would this messages need to look like? What type of information would you prefer – text, videos, images etc.? Would you prefer formal or informal tone in the communication? Would you prefer one-way messages (i.e. only receiving messages) or two-way communication (e.g. having to or preferring to respond)?

**Closing**

Allow me to do a brief recap of the main points. Please do let me know if you think that something has been left out.

Is there anything else you would like to add on the topics we have discussed today?

Thank you very much for participating in today’s discussion and for your valuable input. Your comments will be very helpful to us in developing an intervention for prevention of diabetes in people at risk of diabetes.
