# Supplementary material for: Public Perceptions of Diabetes, Healthy Living, and Conversational Agents in Singapore: Needs Assessment
Source: JMIR Form Res. 2021 Nov 11;5(11):e30435. doi: 10.2196/30435 (PMC8663498; doi:10.2196/30435)
Supplement: Multimedia Appendix 3 [file formative_v5i11e30435_app3.docx]

Multimedia Appendix 2. Themes and subthemes generated from qualitative analysis

| Themes | Sub-themes | Codes | Quotes |
| --- | --- | --- | --- |
| Theme 1: Use of conversational agents | | | |
|  | Defining conversational agents | AI programme/ Virtual call centre | P20 “A chatbot is an AI programme”  P14 “Chatbot is someone, is a virtual call centre that they always like to ask you how can I help you?” |
|  |  | Readily available | P12 “The fact there is readily available information that’s feedback to me in a very short period of time. |
|  |  | Unfamiliarity | P11 “Chat bots, not really in particular.”  P19 “No, never heard of that.” |
|  |  | Usage applications | car insurance, public transport, online shopping, banking and airline services |
|  |  |  |  |
|  | Beneficial features & suggestions for future interventions | [Delivery format:]   - Interactive - Visual aids - Text | P14 “it’s very interactive. And everybody, by talking to them, we get some point, really. So, we enjoy it.”  P12 “I think the use of multimedia here will be best, if there can be images and videos supported by some further text is the user chooses to click on and read more, that will help.” |
|  |  | Pre-prepared options | P20 “was using the IBM Watson kind of AI technology. But it wasn't very, very smart, to say. So I guess if, in this case, it's more of an MCQ kind of thing, like it gives you fixed responses to give” |
|  |  | Group component | P05 “If I did it with other people, like with friends and stuff yea, you could make it like a group effort. Then it’ll be easier because then there are other people to monitor you, just for yourself.” |
|  |  | Novelty | P15 “But that one was not very good because they cannot... It becomes a pattern. Once they get used to it, then people will try to run away from the pattern.” |
|  |  | Appropriate tone to suit function | P19 “I'd prefer a bit of informal, but not too informal, probably a balance.” |
|  |  | Favourable Frequency and duration of interaction | P19 “I'd say two to three times a week. Probably 15, 20 minutes.” |
|  |  | Bidirectional communication | P19 “Preferably two way so that you can at least be assured that your questions are answered.” |
|  |  |  |  |
|  | Unfavourable traits | Incapable of answering questions | P09 “When I had questions, they were not answered appropriately. So, I had to resort to calling them.” |
|  |  | Irrelevant answers | P14 “not useful in answer to our questions. they can't answer specifically to our needs.” |
|  |  |  |  |
| Theme 2: Ubiquity of smartphone application | | | |
|  | Usage of smartphone apps | Messaging apps | WhatsApp, Facebook Messenger, Telegram, WeChat and LINE |
|  |  | Health apps | Step trackers, Healthy 365, my fitness pal, AIA vitality, Fitbit, Samsung Health, HPB (Health Promotion Board) app, Nike Run Club, Chronometer (monitor nutrition) and Stride (habit tracker) |
|  |  |  |  |
|  | Beneficial features | [Positive user experience:]   - ease of use - engaging - multifunctional | P17 “Generally, they are quite convenient “  P18 “Telegram, because of the you can use stickers. The fun part if that it’s engaging, when we message our friends.”  P19 “You get to communicate with someone. You get to video call them as well, so I’d say it’s a very good platform.” |
|  |  | Fulfil a necessity | P15 “I have to use them.” |
|  |  | Provide incentives | P17 “And then for the Healthy 365 app by HPB, I use it to redeem the rewards and participate in their promotional activities and programmes.” |
|  |  |  |  |
|  | Limitations of smartphone apps | Questionable accuracy of content | P07 “I think it’s like a good indicator at least you have some number that you can see. But the accuracy is still debatable.” |
|  |  | Overwhelming frequency | P06 “It can be useful. But it also can be it can also be an harassment (laughs) turns out how you look at it”  “As in because the messages comes so easily so sometimes there’s like a lot of messages to see a day yep.” |
|  |  |  |  |
| Theme 3: Understanding of diabetes | | | |
|  | Defining type 2 diabetes | - elevated blood sugar, - insulin, - reduce sugar intake, - no cure, - medication | P20 “diabetes is a condition in which your sugar in your body is elevated. |
|  |  | Knowledge of its implications on one’s life | P14 “Having good health to be drained away. And then, how does this affect families concerned” “... I was told that people with diabetes may not be able to exercise actively. Have to change the type of exercise that they do. Sometimes breathing will be affected, also. The diets, will have to need a repair. What they like to eat has to change to new diet requirement” |
|  |  | Knowledge of its implications on one’s health | P20 “It affects your vessels in your eyes, your feet and your kidneys, yes, all parts of your body.” |
|  |  |  |  |
|  | Defining prediabetes | - borderline diabetes risk, - reversible, - unfamiliarity with the term | P15 “what I understand that prediabetes is, is that they are closing towards the diabetic range when they do their tests. So they are the borderline testing.”  P17 “It’s very much preventable and treatable before it goes into the diabetes stage.”  P19” To be honest I'm not too sure what that term is.” |
|  |  | Recognition of unequal awareness compared with diabetes | P07 “I think diabetes is more commonly spoken about I think.” |
|  |  |  |  |
|  | Sources of information | Personal experience | P08 “For diabetes right, for my mum, she doesn’t need those injection like insulin. So far its just purely on medication so when I did the test you know like my dietary and also my wellness and so forth right” |
|  |  | Government influence | P14 “The government is trying to educate the citizens about the pre-diabetic issue.” |
|  |  | Lack of information available (in general, to older population) | P12 “not a lot of information readily available”  P09 “Yea, I wish I knew more.” |
|  |  | Additional suggestions (media, internet, hawker centres | P08 “and also there could be more awareness like you know when we go to restaurant or maybe hawkers more information could share like the calories. Because yea I know some hawker centres they do show but it didn’t exactly show the number of calories for all the food.”  P17 “And also, maybe through TV or some of the activities that have been going on, for example, the 10k walk.” |
|  |  |  |  |
|  | Knowledge gaps | How to make healthy lifestyle changes | P11 “In terms of exercise, regulating diet, maybe sleep and health in general.” |
|  |  | Symptoms and prevention of diabetes | P18 “Maybe share with us on symptoms of prediabetes and also give us advice on how to prevent that.” |
|  |  | Awareness of being at risk of diabetes | P09 “What are the risk factors that we have to look out for? Be aware of?” |
|  |  | Application of knowledge gained | P20 “I wouldn’t say you need to learn about it. You just need to practice it and incorporate it into your life.” |
|  |  |  |  |
| Theme 4: Barriers and facilitators to a healthy living in Singapore | | | |
|  | What influences lifestyle choices | Convenience | P20 “Convenience. For example, if I'm studying here then I've one hour to go eat lunch, then usually the places I choose is they have to be nearby.” |
|  |  | Cost | P17 “The biggest problem we have here is the price of healthy food. It’s just infinitely more costly. For example, some salad can cost you six, $7, whereas chicken rice can cost you $3. You cannot possibly eat salad every day even if you are earning okay. But if you are one of those that are low income, definitely you will go for three-dollar chicken rice every day. And in the long run, it just causes a lot of problems.” |
|  |  | Personal preferences | P07 “I mean, I think naturally it will be. I think people naturally gravitate towards like your favourite food” |
|  |  | Social activities | P06 “So sometimes it’s a social setup. Like you go out with friends then and you go for a buffet, so that needs a lot of self-control“ |
|  |  | Advertising & Marketing | P17 “It just depends on what I feel like eating or maybe a bit of marketing as well. For example, if McDonald’s has a new menu, then I might just go for it.” |
|  |  | Nutritional value of food | P20 “then the dietary value of the food also. So how healthy is it and whether it's nutritious. |
|  |  | State of mind | P09 “Sometimes you just forget! Sometimes you just forget and you know, okay lah tomorrow”  “Mood, cravings.” |
|  |  |  |  |
|  | Barriers to healthy living | Dining out | P18 “It's the availability of the ingredients. If you go to hawker centres, I don’t think they really have brown rice. The food is oily, so sometimes you've no choice. Or for drinks, we can’t reduce the sugar intake” |
|  |  | Lack of appeal | P06 “Of course like sometimes healthy food don’t really looks the most palatable” |
|  |  | Health condition | P15 “For me, in the past, because of my brain tumour, I have compulsive eating problems” |
|  |  | Fear of injury | P14 “, but there is also a fear that at a certain age. there is some wear and tear in the body” |
|  |  | Lack of free time | P08 “whether I have time to dock off for a run or not. It really depends because most of the time, I’m actually working quite late…. I think for myself would definitely be work stress…. So myself sometimes I work 15 to 16 hours a day” |
|  |  | Weather limitations | P08 “especially with the haze now even if I do wish I could go run, I can’t.” |
|  |  | Specific events | P14 “where there's some unexpected incidents happen, the stress can pop up.” |
|  |  | Lack of knowledge | P12 “What would help, I don’t really know. I guess if I know I would be able to reduce it.” |
|  |  | Affordability | P18 “Maybe I'd join Zumba. I like that kind of thing but I'm thinking about affordability and the convenience.” |
|  |  |  |  |
|  | Facilitators to healthy living | Early intervention + knowledge building | P05 “It's best to not just educate the older people, because we tend to think that the older people that they will be diabetic easier, so it's best to teach younger ones. “ |
|  |  | Empowering oneself | P05 - “So I have a treadmill at home and I use that. Like I walk as I’m doing some work on my laptop. So I try to I try to incorporate that. Or I might do maybe like 10/15 minutes of of yoga or cardio or something just around the house yea wherever I can.” – (P05) |
|  |  | Moderation | P15 “Stop binge eating. not to have too much of every type.” |
|  |  | Discipline | P19 “I definitely would agree, especially when in Singapore it is food paradise, you actually get good food. I would say it's definitely very hard to maintain that, but with a bit of discipline it should be fine.” |
|  |  | Calorie counting | P17 “I think it does. If I look at the menu item and then it looks like this is 600 calories, which is top of the list out of the whole list of things that I’m looking at, I would think twice about eating it” |
|  |  | Internal motivators | P18 “I just think of wanting to be healthy, so tend to discipline myself on my food intake, so that’s how I do it.” |
|  |  | Reminders | P14 - “So, maybe when it comes to before lunchtime, two hours, tell them this is a suggested meal, then it’s a guideline. Because everybody like to hold their phone, and then the message pops up, and they say, oh, yes, why not, I go and try” |
|  |  | Reduce sitting time | P17 “I will just stand up for longer after a meal rather than sitting down.” |
|  |  | Group support | P16 “I join an exercise group.” |
|  |  | Efficient exercising (less time, more yield) | P20 - “So, yes, I only exercise 15 minutes at each time. But I try to keep it more vigorous. So, yes, I do HIIT kind of training. So I do that about four or five times a week maybe.” “I think that there's more yield in doing vigorous exercise over moderate exercise. So in terms of the efficiency, the amount of time you need to spend, so I try to do more of HIIT.” |
